# Trade-Offs and Synergies Among Grassland Functions: A Case Study in the Three River Headwaters Region

**DOI:** 10.3390/biology15201824

**Published:** 2026-10-13

**Authors:** Wenjing Li, Shikui Dong, Wenlong Li, Wenying Wang

**Affiliations:** 1 College of Geography Science, Qinghai Normal University, Xining 810008, China; 2 School of Grassland Science, Beijing Forestry University, Beijing 100083, China; 3 College of Pastoral Agriculture Science and Technology, Lanzhou University, Lanzhou 730030, China; 4 Academy of Plateau Science and Sustainability, Qinghai Provincial People’s Government & Beijing Normal University, Xining 810008, China

**Keywords:** trade-offs and synergies, grassland, production–living–ecological functions (PLEFs), sustainable development, Three River Headwaters Region

## Abstract

Conflicts among the “production–living–ecological” functions (PLEFs) of grasslands significantly constrain regional economic, social, and ecological security. Therefore, scientifically quantifying and clarifying the trade-offs and synergies among grassland PLEFs in the Three River Headwaters Region (TRHR) is particularly crucial for promoting the realization of regional sustainable development goals. Based on data from 2000 to 2023, this study constructed a grassland PLEFs evaluation index system covering the natural, economic, and social security dimensions of the TRHR. The research calculated the grassland PLEFs and their total functional values, analyzing the spatiotemporal evolution of trade-offs and synergies among these functions. The results indicate significant regional differences in the spatial distribution of grassland PLEFs within the TRHR, with all functions exhibiting an overall upward trend, and the total function is ultimately improved by 35.97%. In addition, against the background of ecological civilization construction, the overall development level of the grassland complex system in the TRHR has continuously improved, and the conflict between pastoral production and living function has been effectively alleviated. However, the conflict between ecological and living function remains a significant bottleneck that constrains the coordinated development of the region. The findings of this study provide a scientific basis for the sustainable use and spatial optimization of grassland resources in the TRHR.

## Simple Summary

Conflicts among the multiple functions of grasslands may threaten regional sustainable development. However, few studies have examined the trade-offs and synergies in the Three River Headwaters Region (TRHR) from the perspective of grassland “production–living–ecological” functions (PLEFs). Based on natural, economic, and social welfare datasets of the TRHR spanning 2000–2023, this study established an evaluation framework for grassland PLEFs. The weighted sum model, correlation coefficient, and bivariate spatial autocorrelation were utilized to calculate grassland PLEFs values and analyze the spatiotemporal evolution of trade-offs and synergies among these functions. The results show that the overall development level of the grassland composite system in the TRHR has continuously increased. Under the background of ecological civilization, the relationship between ecological function and production function in the TRHR is mainly synergy, while the relationship between ecological function and living function is dominated by trade-off. The relationship between production function and living function has shifted from trade-off to synergy. These findings improve our understanding of the interactions among multiple grassland functions in the TRHR and have practical significance for establishing a framework for grassland ecological conservation in the region.

## 1. Introduction

Grassland is an important component of the global ecosystem, covering 20% to 47% of the world’s land surface [1,2]. The report of the FAO indicates that permanent grasslands and pastures cover an area of 3.4 billion hectares, accounting for 69% of the world’s agricultural land [3]. These grassland systems sustain the livelihoods of approximately 800 to 1 billion people worldwide and provide diverse ecosystem services that are crucial to human well-being [4,5]. They maintain ecological integrity, support livestock production, ensure the livelihoods of herdsmen and pass on grassland cultural heritage [6]. However, grassland degradation has become a widely concerned issue: approximately 50% of the world’s grasslands show obvious signs of degradation [7]. Population growth and the continuous rise in demand for livestock products have further exacerbated the overuse of grassland, causing structural damage and functional degradation. The contradiction between grassland ecological protection and economic development has become increasingly prominent [8], leading to the degradation of ecosystem services, the poverty of herders and the decline of human well-being [9]. Such conflicts largely stem from the insufficient coordination of grassland functions and the poor management of their trade-off and synergy relationships [10]. Therefore, sustainable grassland management needs to strike a balance among ecological protection, stable livestock supply, and livelihood security for herdsmen. Research on the multi-functionality of grasslands is of vital importance for maintaining national ecological security, promoting regional economic development, and improving the living standards of herdsmen [11].

The grassland ecosystem is an integrated socio-ecological system providing production, living, and ecological functions (PLEFs) [12,13]. Due to the spatial interdependence of these functions and the diverse interventions of stakeholders, PLEFs exhibit complex trade-offs and synergistic relationships shaped by natural factors, socioeconomic development, and ecological policies [5,6,14,15], which can lead to ecosystem imbalances [16]. Additionally, the spatiotemporal evolution and structural changes in PLEFs lead to differing stakeholder perceptions of trade-offs and synergies [17]. It is worth noting that changes in one grassland function will affect the others, thereby influencing the overall ecosystem as well as the coordination and sustainability among functions [18]. Overemphasizing a single function often sacrifices others, exacerbating trade-offs and functional conflicts, whereas strengthening synergies promotes functional integration and ecosystem coordination [12]. Additionally, the status of grassland PLEFs determines the health of regional grassland ecosystems and affects population capacity and livestock production [19]. The current imbalance among the three functions has led to grassland degradation and unsustainability [19]; without effective measures, a vicious cycle of “ecological disaster-production collapse-decline in living standards” will emerge. Therefore, studying the trade-offs and synergies among grassland PLEFs is particularly important for resolving conflicts in multi-objective grassland management.

The Three River Headwaters Region (TRHR), as the lifeline of China’s ecological security and the master switch of Asian water systems, holds extremely significant ecological importance and has a profound impact on China’s sustainable development and global ecological security. Animal husbandry dominates local production, and grassland resources support the livelihoods of 70% of the regional population [20]. However, driven by human disturbances such as climate change, overgrazing, resource exploitation, and engineering construction, the grassland in the TRHR is undergoing degradation and its ecological processes are being disrupted. Degraded grassland accounts for 26% to 46% of the total grassland area, threatening the sustainability of local biodiversity, grassland resources and pastoral production [21,22]. In the context of ecological civilization, the government has implemented a series of restoration projects in the TRHR, including grassland restoration, vegetation reconstruction, grazing bans and livestock reduction measures, to slow down grassland degradation. Although these policies have alleviated the grazing pressure, they have also affected the balance among the PLEFs of the grasslands. Strict grazing bans may weaken the self-recovery capacity of some grasslands and reduce the income of herders by limiting the number of livestock. From a sustainability perspective, such policy interventions may disrupt the interactions among PLEFs, posing a challenge to the long-term sustainability of grassland systems.

The interactions among grassland PLEFs exhibit non-linear, multi-coupled, multi-dimensional cascading effects and feedback mechanisms [5]. This complexity hinders systematic understanding of functional relationships and impedes the design of coordinated conservation-development pathways [14,23,24]. It is therefore urgent to quantify the dynamic trade-offs and synergies among grassland PLEFs to reconcile human well-being improvement and ecosystem conservation. However, most studies focus on grassland ecosystem services and their pairwise trade-offs and synergies [18,25,26], while other research addresses how soil properties, plant traits, and microbial activity regulate grassland health and functional evolution at local scales [27]. Adopting a more comprehensive analytical framework that emphasizes grassland PLEFs helps us better understand the interactions between grassland ecology and human activities in the TRHR under pastoral practices and ecological policy interventions [28]. Accordingly, this study established a multi-dimensional evaluation system covering ecological, economic, and social security for grassland PLEFs. We quantitatively assess spatiotemporal variations in TRHR grassland PLEFs from 2000 to 2023 and analyzed the trade-offs and synergistic changes across different time periods.

## 2. Materials and Methods

### 2.1. Study Area

**Figure 1:**
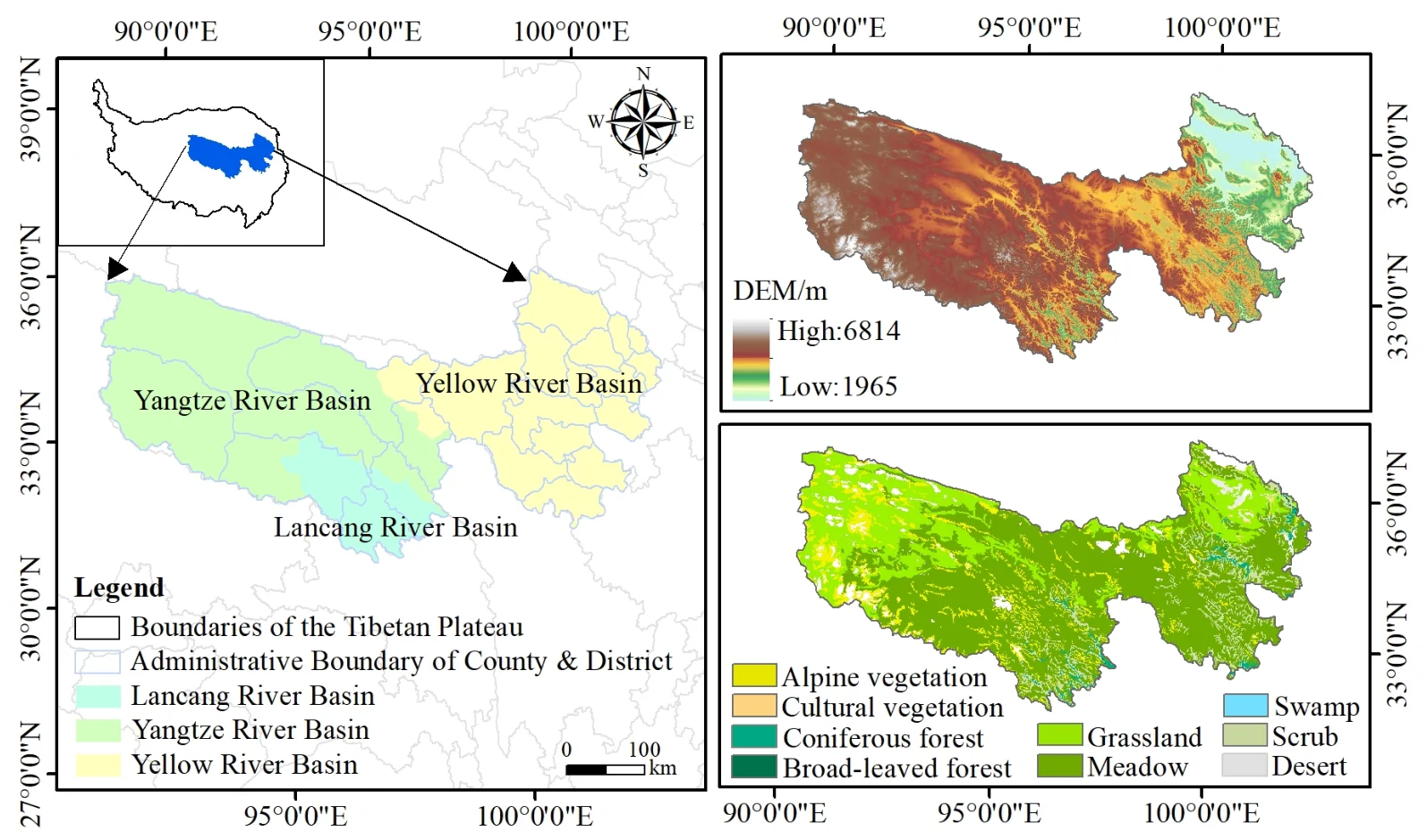
Study area location, elevation, and vegetation types (This map has no copyright issues).

The TRHR is situated in the central region of the Tibetan Plateau, specifically in the southern part of Qinghai Province (Figure 1). This area constitutes 3.65% of the entire land mass of China and 50.43% of Qinghai itself. It includes 16 counties and one township (Tangula). The average altitude within the TRHR exceeds 4000 m, contributing to its distinct environmental conditions. The TRHR features a typical plateau continental climate. The area experiences intense annual solar radiation, long sunshine duration, and ample sunlight throughout the year, ranging from 2300 to 2900 h. The region also has significant diurnal and annual temperature variations, with the yearly average temperature is −3.6 °C, and the temperature decreases from the southeast to the northwest. Regarding precipitation, annual rainfall totals between 200 and 800 mm, while evaporation rates lie between 730 and 1700 mm [29]. These climatic features together shape the ecosystem and living circumstances within the TRHR.

Alpine grasslands and meadows are the most widespread ecosystem types in this area, covering 24% and 58% of the area, respectively [30]. Significant variations in types of vegetation are present between the eastern and western sections of the TRHR. Specifically, grasslands diminish from east to west, while bare soil predominantly covers the northwest, comprising 33.10% of the land area. Livestock husbandry is the main industry in the TRHR. The eastern region is an agro-pastoral transitional zone, while the western region is the uninhabited Hoh Xil area.

### 2.2. Data

This study utilizes vegetation, meteorological, and socioeconomic data spanning from 2000 to 2023 (Table 1). Among them, the remote sensing data undergo preprocessing such as projection, resampling and cropping to generate raster data with a resolution of 1 km covering the study area. Due to the lack of more detailed statistical data in the study area, socio-economic data were calculated at the county level and then resampled to the corresponding 1 km raster units of each county.

Based on the major ecological protection policies, construction projects, and institutional reforms of the TRHR, we have selected six key time points: In 2000 (the national nature reserve was established), in 2005 (the first phase of the ecological protection and construction project was initiated), in 2010 (the Implementation Measures for the Ecological Compensation Mechanism was promulgated), in 2015 (designated as a pilot area for the national park system reform), in 2020 (the second phase of the ecological protection and construction project was completed), and in 2023 (current situation).

### 2.3. Establishment of the Grassland PLEFs Evaluation System in the TRHR

To impartially assess the trade-offs and synergies of grassland PLEFs in the TRHR, we developed a comprehensive evaluation system (Table 2). Based on the characteristics of the grassland ecosystems in the TRHR and the specific features of the area, this framework integrates natural, economic, and social welfare factors by leveraging existing research findings [5,31,32]. In selecting indicators, we prioritized data availability and scientific validity to avoid bias and subjectivity, ultimately identifying 17 key indicators.

The ecological functions of grasslands refer to their habitat, biological characteristics, or ecosystem processes [33]. They provide life-support and environmental regulation functions, serve as the foundation for sustaining social and economic development, and are of public welfare attributes [9]. Grassland production function refers to the provision of various consumable resources for living systems. It represents a commodifiable function with economic value [9]. Beyond its roles as an ecological security barrier and provider of livestock production functions, grasslands sustain unique living and production modes adapted to specific environmental conditions [34]. Accordingly, the indicators selected in this study capture not only the direct supporting functions of grasslands, but also their derived outputs and grassland-dependent human well-being. Among these, the production function (PF) of grasslands provides the resources necessary for developing livestock husbandry and supporting regional economic growth [35]. The selected indicators include grass yield, livestock carrying capacity per unit area, total meat production, and other factors. These indicators characterize the PF of grasslands from multiple dimensions, including grassland supply, carrying capacity, and physical livestock output. The living function (LF) reflects the social attributes of the grassland [19]. We employed indicators such as per capita GDP, the number of hospital beds, the number of students in school, and the number of social welfare institutions to characterize the income level and public service accessibility of grassland-dependent herdsmen, reflecting the ability of grasslands to maintain the survival and settlement of pastoral communities [31]. The ecological function (EF) encompass a range of ecosystem services, such as water conservation, climate regulation, carbon sequestration and oxygen release, soil retention and air purification [11,36]. Key indicators include climate, vegetation, atmospheric quality, and soil–water conservation. Given that TRHR showed a warm and humid trend during the research period, the increase in temperature and precipitation promoted vegetation growth [36]. Therefore, the annual average temperature and annual precipitation are defined as positive ecological indicators.

### 2.4. Comprehensive Assessment Model for Grassland EPLFs in the TRHR

All indicators were normalized to [0, 1] via extreme value normalization using the global maximum and minimum values during 2000–2023. To improve weighting robustness, we adopted a combined AHP-entropy weighting approach, with all calculations performed in R version 4.4.2. For AHP subjective weighting, pairwise comparison matrices were established based on indicator relative importance, and all matrices satisfied the consistency test with a consistency ratio CR < 0.1. For entropy objective weighting, standardized indicator data were used to calculate objective weights. The final comprehensive weights of each indicator are obtained by combining the AHP weight with the entropy weight in equal importance:

(1) $$w_{i}=0.5w_{{\mathrm{AHP}}_{i}}+0.5w_{{\mathrm{entropy}}_{i}}$$

where *w_i_* is the comprehensive weight, $w_{{\mathrm{AHP}}_{i}}$ and $w_{{\mathrm{entropy}}_{i}}$ represent the AHP subjective weight and entropy objective weight of indicator *i*, respectively.

Finally, the values of grassland PLEFs and the total function value were calculated according to the following formula:

(2) $$F_{i}=\sum_{i=1}^{n}\;W_{i}\times S_{i}$$

where *F_i_* is the functional value of the grassland function, *S_ij_* is the standardized value of the index.

### 2.5. Evaluation of Trade-Offs and Synergies Among Grassland PLEFs in the TRHR

At the regional scale, Spearman’s rank correlation analysis was performed using R software to quantify the overall relationships among grassland PLEFs. The specific formula is as follows:

(3) $$\rho =1-\frac{6\sum d_{i}^{2}}{n\left(n^{2}-1\right)}$$

where *ρ* is Spearman’s rank correlation coefficient; *d_i_* is the rank difference between the two variables for the *i*th sample; and *n* is the number of samples. *ρ* > 0 indicates a synergistic relationship, whereas *ρ* < 0 indicates a trade-off relationship.

To further reveal the local spatial disparities of trade-off and synergy relationships among grassland PLEFs, a bivariate local Moran’s I analysis was applied [37]. Compared with univariate analysis, which focuses solely on the spatial characteristics of a single function, this method extends across different functional dimensions and incorporates the spatial distribution data of both grassland functions into a unified analytical framework. In this study, 1 km × 1 km grid-based data extracted by ArcGIS 10.8 were imported into GeoDa 1.22.0 to calculate the global bivariate Moran’s I and generate local LISA cluster maps. The calculation formula is as follows:

(4) $$I=\frac{F_{i}^{a}-{\bar{F}}_{a}}{S^{a}}\sum_{j=1}^{n}\;\left[W_{\mathrm{ij}}\frac{F_{j}^{b}-{\bar{F}}_{b}}{S^{b}}\right]$$

where $F_{i}^{a}$ represents the functional value of region *i*, $F_{j}^{b}$ represents the functional value of region *j*, ${\bar{F}}_{a}$ and ${\bar{F}}_{b}$ represent the mean values of the k-th and i-th functions, respectively, and *S^a^*, *S^b^* represent the variances of items a and b, respectively. In our calculations, we constructed a first-order Rook contiguity matrix and used a permutation test with 999 random permutations to assess its significance. We classified trade-off and synergy patterns as HH (high–high synergy), LL (low–low synergy), HL (high–low trade-off), LH (low–high trade-off), and NN (non-significant) regions.

## 3. Results

### 3.1. Evaluation Results of Grassland PLEFs in the TRHR

**Figure 2:**
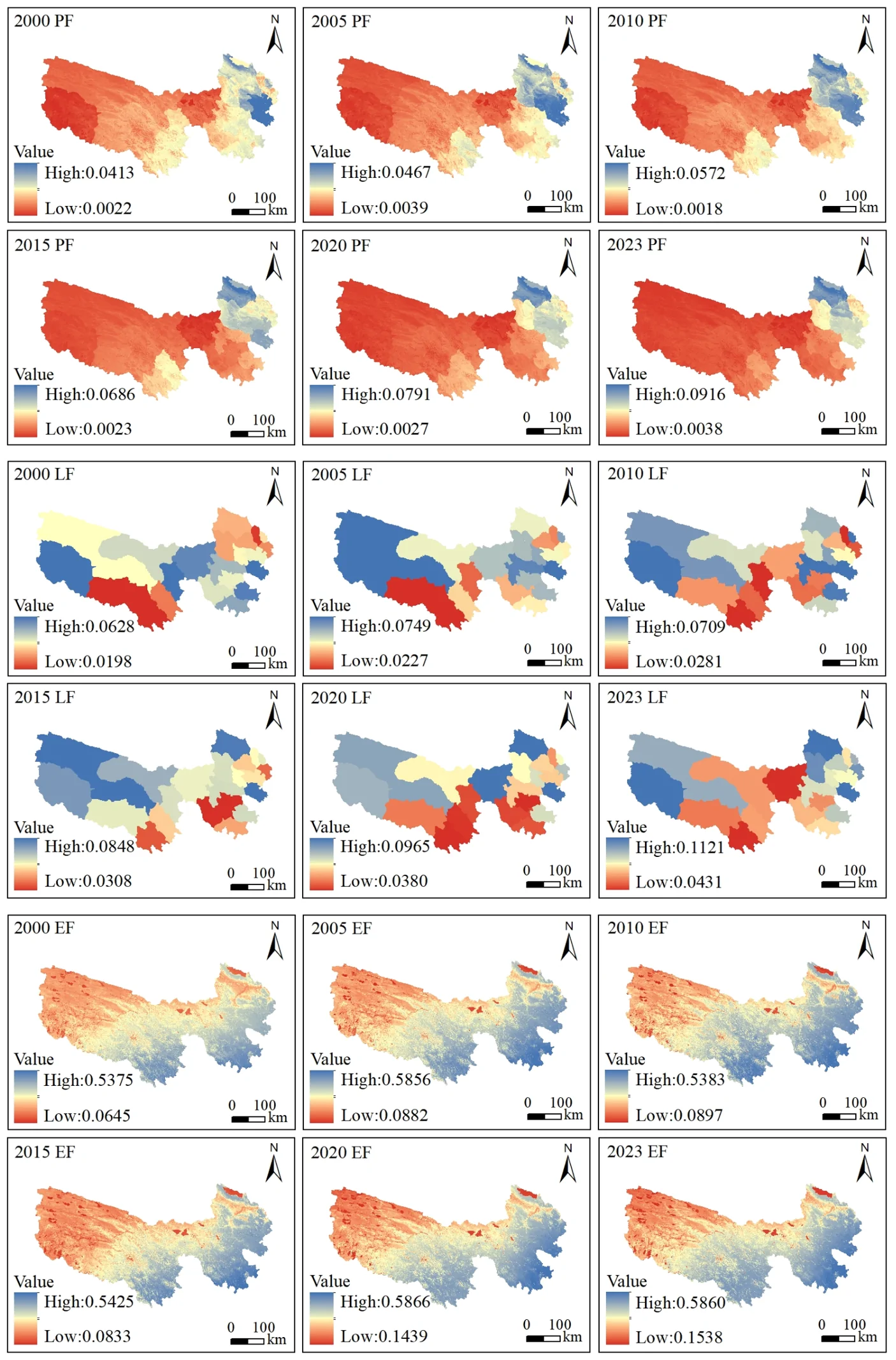
Distribution of grassland PLEFs in the TRHR.

Based on the evaluation indicator system, we calculated the average values of PF, LF, EF, and TF for grasslands in the TRHR for the six time periods of 2000, 2005, 2010, 2015, 2020, and 2023 (Table 3). Additionally, a map showing the spatial distribution of grassland PLEFs in the TRHR was created (Figure 2).

From a temporal perspective (Table 3), the grassland PLEFs in the TRHR exhibited an upward trend from 2000 to 2023. Specifically, the PF and the LF showed a consistent increase, while the EF followed a pattern of increase, decrease, and subsequent increase, culminating in an overall increase of 35.97% for the TF. In detail, PF increased by 16.18%, 27.94%, 36.03%, 40.44%, and 48.53% across each phase; LF increased by 10.64%, 23.51%, 48.02%, 66.09%, and 67.08%; and EF saw increments of 13.52%, 9.60%, 0.17%, 30.08%, and 31.04%, respectively.

From a spatial perspective (Figure 2), significant regional differences exist in the distribution of grassland PLEFs within the TRHR. Specifically, the PF demonstrates a spatial pattern with higher values in the northeast and lower values in the central and northwestern areas. This distribution is primarily attributable to greater forage production and higher levels of economic development in the northeast, which in turn results in elevated PF values. The LF displays the most pronounced regional variation in spatial distribution, with notable changes across different stages, this variation is largely associated with disparities in herders’ economic income and the quality of living services and support across various counties. Spatially, the EF reveals a distribution pattern characterized by high values in the southeast and low values in the northwest. This phenomenon is due to the southeast’s high vegetation cover, favorable water and heat conditions, and robust water conservation and carbon sequestration capabilities, while the northwest is marked by high elevation, low vegetation cover, extensive bare ground, poor water and heat conditions, and a relatively fragile ecosystem.

Due to variations in economic development, social services, and the ecological environment across different time periods and within the TRHR, the spatiotemporal heterogeneity of grassland PLEFs in this area is notably significant. In 2000, the region experienced low levels of economic development and basic infrastructure, coupled with severe grassland degradation, resulting in the lowest levels of grassland PLEFs. However, beginning in 2005, rapid socioeconomic development, alongside the ongoing implementation of ecological conservation projects and compensation measures, led to a gradual increase in grassland PLEFs in the TRHR, which peaked by 2023.

### 3.2. Trade-Offs and Synergies Among Grassland PLEFs at the Regional Scale

**Figure 3:**
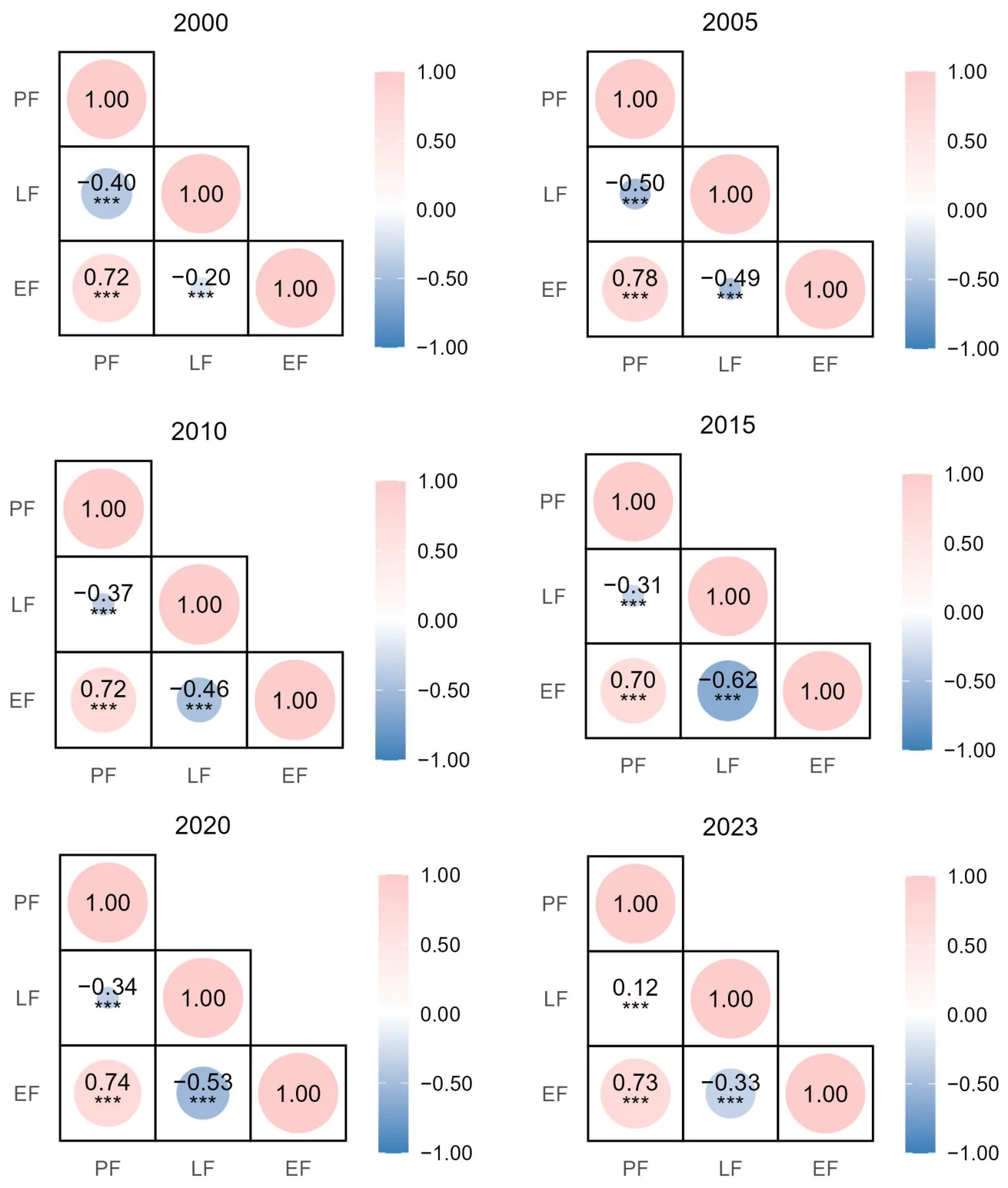
Trade-offs and synergies among grassland PLEFs, 2000–2023. (*** *p* < 0.001)

Figure 3 illustrates the trade-offs and synergies among the three grassland functions at the regional scale in the TRHR, with all correlation coefficients passing the significance test at the 0.001 level. As shown in the figure, the correlation coefficients among grassland PLEFs fluctuated to varying degrees across different years, but exhibited no obvious monotonic increasing or decreasing trend, indicating that the interactions among these functions are dynamic. During 2000–2023, trade-offs were consistently observed between the ecological and living functions, with the strongest trade-off intensity of −0.62 occurring in 2015; synergies were consistently found between the ecological and production functions, with correlation coefficients remaining above 0.70; and the relationship between the production and living functions shifted from trade-offs to synergies.

### 3.3. Trade-Offs and Synergies Analysis of Grasslands’ Functional Pairs

To investigate the trade-offs and synergies between any two functions in the TRHR from 2000 to 2023, we used Formula (4) to calculate the global bivariate Moran’s I for different pairs of functions in the region. The results passed the significance level test at 0.05 (Table 4).

**Figure 4:**
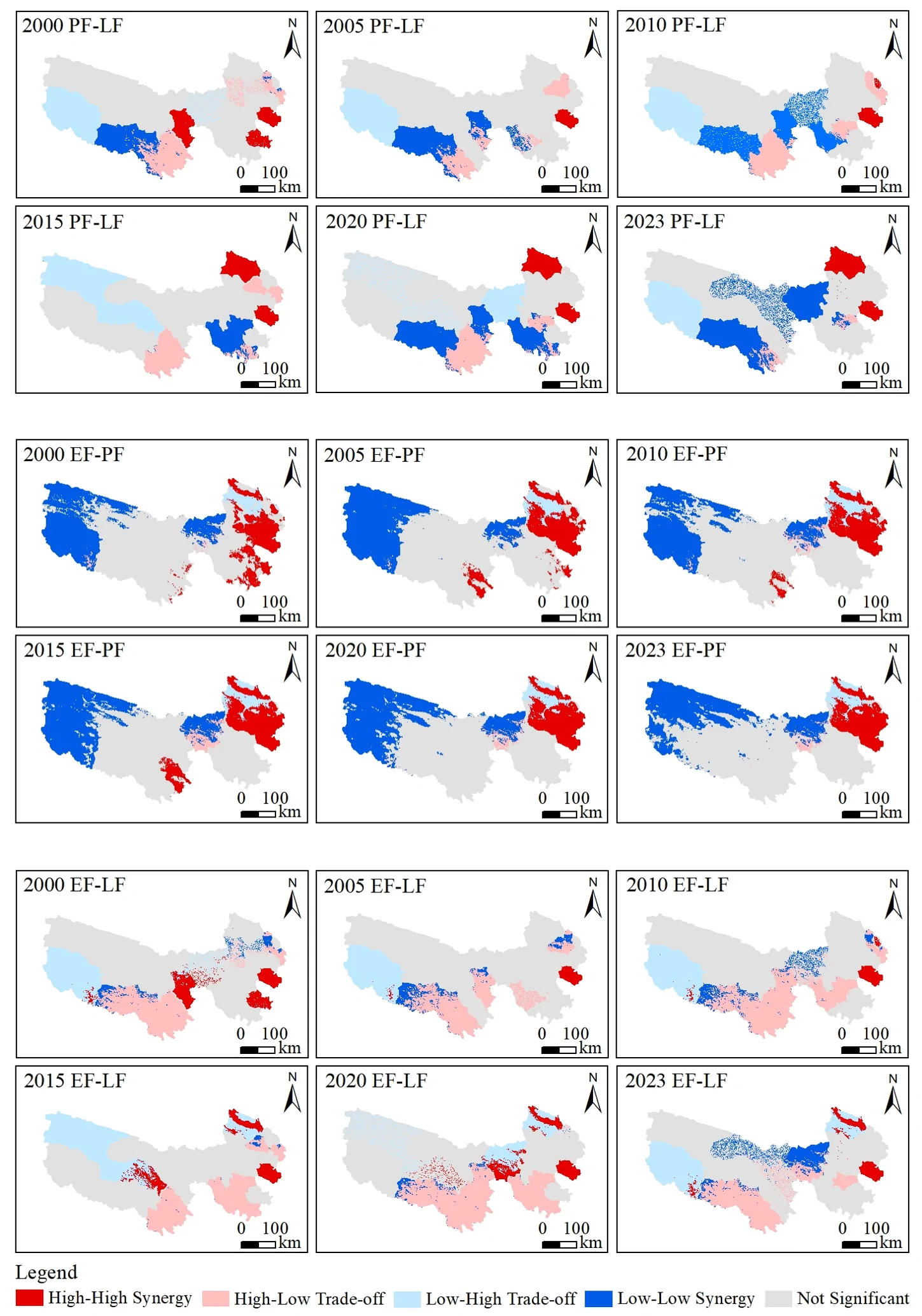
Spatiotemporal distribution of trade-off and synergistic relationships among grassland PLEFs, 2000–2023.

From a spatial perspective (Figure 4), the distribution of trade-offs and synergies between the PF-LF of grasslands in the TRHR exhibited significant fluctuations from 2000 to 2023. Notably, only the Henan County area consistently maintained an HH-type synergy. On average, synergy-type areas comprised approximately 19.11%, while trade-off-type areas constituted approximately 22.91%. The relationship between EF-PF exhibited minimal variation, with synergies prevailing and consistently accounting for over 36% of the total area. Among these, HH-type synergistic areas are predominantly located in the eastern region, where the northeastern part remains relatively stable, while the southeastern part experiences significant fluctuations. LL-type synergistic areas are found in the western region, specifically in Tanggula Town, the northwestern part of Zhiduo County, and the northern part of Maduo County. HL-type trade-off areas are scattered throughout Maduo County, whereas LH-type areas are primarily concentrated in Gonghe County. The relationship between EF-LF is characterized by trade-off types (HL, LH), with synergistic and trade-off regions comprising 8.28% and 33.72%, respectively. Spatial stability is relatively low; only Nangqian County (HL type) and Henan County (HH type) exhibited consistency.

**Figure 5:**
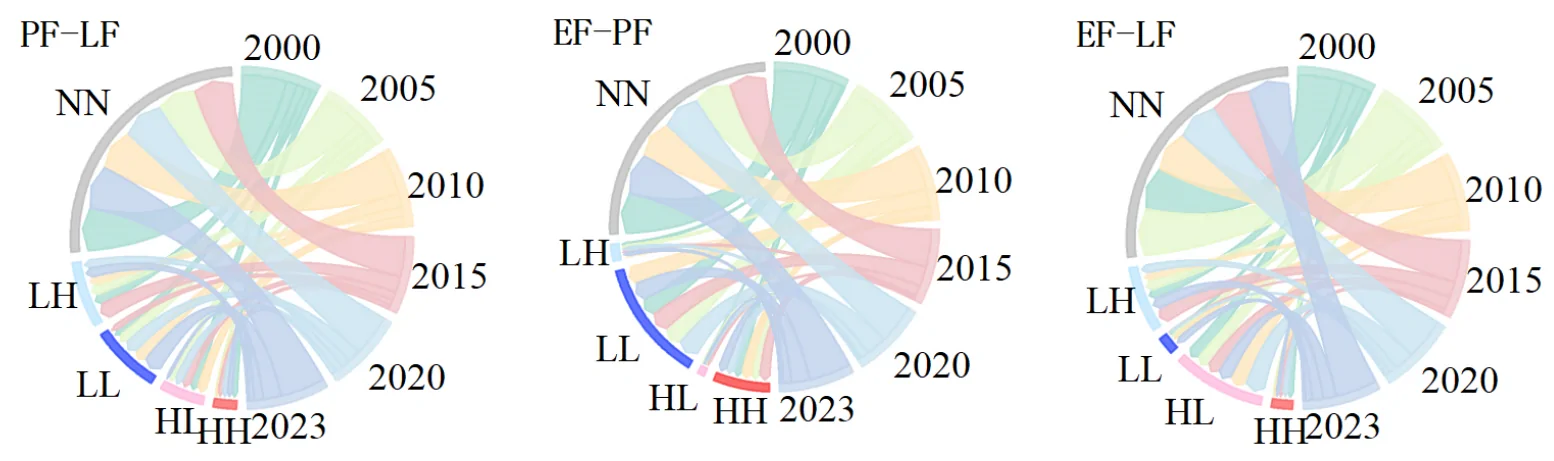
Changes in the number of trade-offs and synergies among grassland PLEFs in the TRHR.

When viewed across different time periods (Figure 5), the changes in the number of trade-offs and synergies between the PF-LF and EF-LF exhibit phased fluctuations. Among these, the fluctuations of PF-LF are more pronounced, with changes in the number of synergies ranging from −6.9% to +10.46%, while the changes in EF-LF are relatively minor. In contrast, the proportion of trade-offs and synergies between the EF-PF remains relatively stable.

Overall, significant differences exist in the spatiotemporal characteristics of trade-offs and synergies among grassland PLEFs in the TRHR. The PF-LF relationship has transitioned from a trade-off to a synergy, while the EF-PF relationship has consistently exhibited a dominant synergy. In contrast, the EF-LF relationship has been characterized predominantly by trade-offs. Furthermore, the trade-offs and synergies in the PF-LF relationship fluctuate dramatically across space, followed by EF-LF, whereas the EF-PF relationship remains relatively stable.

## 4. Discussion

### 4.1. Changes in Trade-Offs and Synergies of Grassland PLEFs in the TRHR

Grasslands produce goods with direct economic value and provide a variety of ecosystem services. Degradation of grassland ecosystems leads to ecological imbalance and negatively impacts social and economic development. It can even seriously damage the production and livelihoods of farmers and herders [38]. Consequently, it is essential to attain the simultaneous objectives of enhancing livelihoods and safeguarding the environment, while successfully managing the trade-offs and synergies between ecological and socioeconomic factors for the sustainable advancement of grassland regions [27]. Nevertheless, the variety in human decisions and choices means that placing too much emphasis on a single function may diminish or even overlook other functions [39]. This conflict between functions intensifies the trade-offs and competition among the various functions of grasslands and may constrain the sustainable development of grasslands [40].

This study analyzed the spatiotemporal dynamics of trade-offs and synergies among grassland PLEFs in the TRHR across different time periods. The study found that the relationship among grassland PLEFs in the region evolved due to varying levels of ecological protection and socioeconomic advancement over time. Between 2000 and 2023, the TRHR underwent a series of ecological conservation initiatives, including the establishment of a national nature reserve, the implementation of Phase I and Phase II ecological conservation projects, and the creation of a national park. During this process, the grassland area in the TRHR was restored and expanded [41], and the grassland’s EF gradually improved. The improvement in grassland resources has also supported the development of livestock industry, thereby boosting the enhancement of grassland PF and resulting in a predominantly synergistic relationship between EF and PF. Meanwhile, ecological conservation policies have reduced the region’s livestock population by 15.34% [22], and traditional livelihood practices of herders have faced certain restrictions, leading to a trade-off between EF and LF. In addition, with the distribution of various ecological protection subsidies and the continuous improvement of mechanisms for realizing the value of ecological products, herders’ livelihoods have become more diversified, which has, to a certain extent, increased their incomes [42].

These findings reflect the combined effects of multiple intertwined factors, including ecological protection policies, socioeconomic development, and social security construction, which jointly shape the spatial distribution and dynamic variations in the trade-offs and synergies of grassland PLEFs under the ecological civilization construction framework. To a certain extent, excessive emphasis on ecological conservation, such as the stringent implementation of grazing prohibition and grass-livestock balance regulation, may potentially reshape the structural composition and interactive relationships of grassland PLEFs. This could limit PF and LF, thereby causing changes in the trade-offs and synergies between livestock production, herders’ livelihoods, and ecological protection [43]. Studies have shown that the grazing activities of different intensities have different trade-offs and synergistic effects on grassland PLEFs. Moderate grazing promotes optimal synergy between grassland functions and maintains the stability and sustainability of grassland ecosystems. Conversely, while fencing enclosures can help restore grasslands and enhance ecosystem service synergy [44], complete grazing prohibition violates ecological laws and can result in the degradation of grassland ecological functions and a decrease in system adaptability [45,46].

### 4.2. Uncertainties and Limitations of Trade-Offs and Synergies Evaluation in the TRHR

Grassland is an important element in ecological protection, and its ecosystem services are jointly regulated by natural conditions and human activities. Under natural conditions, the multi-functionality of grasslands is mainly driven by competitive relationships rather than trade-offs [47]. In contrast, human intervention, especially in livestock production and pastoral area utilization, has significantly reshaped the interaction among the multiple functions of grassland and triggered complex trade-offs and synergies [48]. Previous studies have confirmed that the ecological cognition and resource utilization preferences of local herdsmen profoundly determine the health status of grassland ecosystems. Especially, excessive human reliance on grassland resources often suppresses the functions of other ecosystems, thereby having adverse effects on the structure, functional integrity, ecological stability and long-term sustainability of alpine grassland systems [41]. Therefore, human preferences and utilization orientations towards grassland ecosystem services are the key driving factors for the trade-off and synergy of grassland PLEFs [47]. Globally, there is an inherent trade-off among livestock production, biodiversity conservation, and the security of residents’ livelihoods in grazing ecosystems [49].

Current research on the multifunctionality of grasslands in the TRHR has mainly focused on quantifying the trade-offs and synergies among ecosystem services [50]. Furthermore, the current assessment of grasslands PLEFs mostly relies on land use classification data and uses construction and cropland to characterize living function [51], failing to fully reflect the direct living support capacity that natural grasslands provide for the population in pastoral areas. Compared with previous studies, this research constructed a comprehensive evaluation framework covering ecological, production, and living functions, and systematically analyzed the trade-off and synergy characteristics of grassland PLEFs in the TRHR. The results show that the trade-off and synergy of grassland PLEFs in the TRHR exhibit significant spatiotemporal heterogeneity. This spatio-temporal difference can be attributed to two major factors: the inherent regional differences in natural conditions and socio-economic development levels within the TRHR, as well as the inconsistency in the implementation effects of ecological protection policies in different periods and regions, which in turn leads to the spatial uncertainty of the distribution of grassland PLEFs trade-offs and synergies.

However, although a comprehensive evaluation system for grassland PLEFs has been established and the trade-offs and synergies among grassland PLEFs in the TRHR have been evaluated, there are still deficiencies in the methods that need to be improved. Firstly, grassland PLEFs are driven by multiple factors such as culture, institutional and ecological policies [52,53], which may lead to potential correlations and information redundancy among evaluation indicators. Secondly, the selection of indicators and the calculation of weights are restricted by data accessibility, spatial resolution, the subjective judgment of researchers, and weighting methods. These factors inevitably introduce evaluation uncertainty and limit the direct comparability of the results of this study with those of similar regional studies [24]. Furthermore, scale selection is crucial for analyzing the spatiotemporal evolution of grassland PLEFs [32,54]. In future research, we will further optimize the indicator system, improve the classification standards, and systematically clarify the intrinsic coupling mechanism among multi-dimensional indicators. At the same time, a comprehensive multi-scheme sensitivity test will be carried out. In addition, higher-resolution datasets and a more comprehensive analytical framework will be adopted for cross-regional comparative analysis to reduce uncertainties arising from scale effects, indicator selection, and weighting schemes.

### 4.3. Policy Implications of Analytical Assessment of Grassland PLEFs and Suggestions for Grassland Management

An analytical assessment of grassland PLEFs is vital for attaining sustainable development. Understanding the interrelationship among grassland PLEFs is crucial for grasping the multifunctionality of grasslands and making scientific decisions [18]. However, striking an effective balance among these functions is currently a key challenge in grassland management [31]. Previous evaluations of the TRHR mainly focused on grassland ecosystems [55,56]. Therefore, this study adopts a comprehensive perspective, integrating factors related to natural, economic and social well-being, and focuses on analyzing the dynamic trade-offs and synergistic interactions among PLEFs in this region, aiming to reveal the complexity and multi-dimensional value of grassland ecosystems.

This study found that grassland PLEFs in the TRHR exhibit significant spatial differentiation characteristics, and there are differences in the trade-offs and synergistic relationships among different functional pairs. These results can provide scientific guidance and decision support for protecting the grassland ecological environment in the TRHR, rationally developing and utilizing grassland resources, optimizing and adjusting grassland protection policies, and promoting a harmonious relationship between humans and nature. Furthermore, these results can contribute to the sustainable management of grasslands. To this end, the study provides recommendations for managing grasslands and protecting the ecology in the TRHR. First, based on the spatial differentiation and overall enhancement characteristics of the grassland PLEFs in the TRHR, differentiated zoning management should be implemented. Adhering to the principle of ecological priority, the spatial configuration of grasslands should be optimized, strengthening the management of ecologically vulnerable areas and coordinating the development of advantageous production-living zones to enhance the comprehensive functions of grasslands. Second, in response to the trade-offs between regional ecology and living functions, precise ecological restoration and the improvement of ecological compensation mechanisms should be promoted. The existing ecological engineering restoration scope is limited and its effects are short-lived [54,55], and it has not fundamentally improved the ecological status of grasslands. Therefore, targeted restoration and the establishment of a diversified compensation system should be prioritized, balancing ecological protection with herders’ livelihoods to promote coordinated regional development. Third, in light of the spatial correlation characteristics of grassland PLEFs, a dynamic and refined grazing monitoring and management system should be constructed. By drawing on advanced management technologies and concepts, a dynamic monitoring network for grazing pressure should be established, along with a long-term mechanism for regional ecological protection [57]. Fourth, based on the collaborative development needs of grassland PLEFs, traditional pastoral models should be optimized [38]. By developing cooperative economies in pastoralism, herders can be guided to transform their production and lifestyle, advancing green ecological pastoralism. Under the premise of strictly adhering to ecological bottom lines, ecological protection and livelihood development should be coordinated to achieve synergistic benefits between the ecological and social outcomes of the TRHR.

## 5. Conclusions

Balancing grassland PLEFs is essential for regional ecological security, stable pastoral livelihoods, and socio-ecological sustainability in the TRHR. This study evaluated the spatiotemporal dynamics and interactions of the grassland PLEFs in the TRHR from 2000 to 2023. The results indicate that all three grassland functions improved during the study period, with a total function improvement of 35.97%. Due to regional differences in natural conditions and levels of socioeconomic development, significant regional variations were observed in the grassland PLEFs of the TRHR. Furthermore, the relationships and changes varied among different pairs of functions. This study systematically assessed the spatiotemporal patterns of grassland multifunctionality in the TRHR from the perspective of grassland PLEFs, providing a basis for decision-making regarding the optimized management and scientific conservation of grassland resources in the region. The study contributes to the development of a new framework for grassland ecological conservation in the TRHR, and its analytical framework also offers insights into research on the multifunctional coupling of grassland systems and the sustainable development of plateau ecological regions.

## Figures and Tables

**Table 1:** Data sources.

Data Type	Data Year	Data Source	Data Description
Climate data	2000–2023	National Earth System Science Data Center	Mean annual temperature and precipitation, 1 km
Net Primary Production (NPP)	2000–2023	Google Earth Engine (GEE)	MODIS, 500 m
Fractional Vegetation Cover (FVC)	2000–2023	National Tibetan Plateau Data	250 m
Soil erosion	2000–2023	Geographic Data Sharing Infrastructure, Global Resources Data Cloud	1 km
PM2.5	2000–2023	National Tibetan Plateau Data	1 km
Socioeconomic data	2000–2023	Statistical Yearbooks and the Bureau of Statistics	Including GDP, population, livestock, etc.

**Table 2:** Evaluation index system and corresponding weights for grassland PLEFs in the TRHR.

System	Subsystem	Indicators and Direction	Units	Weight
Total Function (TF)	Production Function (PF)	Grass yield (+)	g·m^−2^	0.0286
		Livestock carrying capacity per unit area (+)	su/ha	0.0219
		Meat production (+)	kg	0.0302
		The added value of the secondary industry (+)	Ten thousand yuan	0.0199
		The added value of the tertiary industry (+)	ten thousand yuan	0.0031
	Living Function (LF)	Per capita GDP (+)	yuan/person	0.0729
		The average number of livestock per person (+)	su/person	0.0355
		Number of students per thousand people in school (+)	person	0.0428
		The number of beds in social welfare adoption units per thousand people (+)	unit	0.0160
		The number of hospital beds per thousand people (+)	unit	0.0374
	Ecological Function (EF)	Mean annual temperature (+)	°C	0.0632
		Mean annual precipitation (+)	mm	0.0407
		NPP (+)	g·m^−2^·a^−1^	0.0674
		FVC (+)	-	0.1300
		Soil erosion (−)	t·km^−2^·a^−1^	0.0272
		PM2.5 (−)	µg/m^3^	0.1350
		Water conservation (+)	mm	0.2283

**Table 3:** Evaluation results of grassland PLEFs from 2000 to 2023.

Function	2000	2005	2010	2015	2020	2023
PF	0.0136	0.0158	0.0174	0.0185	0.0191	0.0202
LF	0.0404	0.0447	0.0499	0.0598	0.0671	0.0675
EF	0.2906	0.3299	0.3185	0.2911	0.3780	0.3808
TF	0.3445	0.3903	0.3857	0.3692	0.4641	0.4684

**Table 4:** Global bivariate Moran’s I from 2000 to 2023.

Function	2000	2005	2010	2015	2020	2023
PF-LF	−0.2763	−0.3242	−0.1826	−0.101	0.0071	0.4912
EF-PF	0.6451	0.6598	0.5524	0.505	0.4252	0.3481
EF-LF	−0.2608	−0.4764	−0.4651	−0.6392	−0.5201	−0.2969

## Data Availability

The original contributions presented in this study are included in the article. Further inquiries can be directed to the corresponding author.

## Abbreviations

The following abbreviations are used in this manuscript:

TRHR	Three River Headwaters Region
PLEFs	Production–living–ecological functions
NPP	Net Primary Production
FVC	Fractional Vegetation Cover
PF	Production function
LF	Living function
EF	Ecological function
HH	High–High type
LL	Low–Low type
HL	High–Low type
LH	Low–High type
NN	Not Significant
